# Hydralazine-Induced Antineutrophilic Cytoplasmic Antibody (ANCA)-Associated Vasculitis Presenting as Crescentic Glomerulonephritis

**DOI:** 10.7759/cureus.59100

**Published:** 2024-04-26

**Authors:** Yoan E Rodriguez, Hovra Zahoor, Kunal Patel, Christian Castro Ruiz, Neda Shahoori

**Affiliations:** 1 Internal Medicine, HCA Florida Orange Park Hospital, Orange Park, USA; 2 Nephrology, HCA Florida Orange Park Hospital, Orange Park, USA

**Keywords:** pauci-immune vasculitis, necrotizing and crescentic glomerulonephritis, small vessel vasculitis, anca-associated vasculitis (aav), hydralazine

## Abstract

Hydralazine is a vasodilator medication commonly used for treating hypertension. While generally well-tolerated, in rare cases it can induce autoimmune reactions, including anti-neutrophil cytoplasmic antibody (ANCA)-associated vasculitis. This case report presents a patient who developed ANCA-associated vasculitis resulting in crescentic glomerulonephritis (CrGN) following long-term hydralazine therapy, highlighting the importance of considering this rare adverse effect in patients with unexplained renal decline.

## Introduction

Hydralazine, a vasodilator used in the treatment of hypertension, is a rare cause of antineutrophil cytoplasmic antibody (ANCA)-associated vasculitis (AAV) with pulmonary and renal involvement [1]. AAV is a pauci-immune inflammatory condition characterized by small vessel necrotizing vasculitis [2]. Crescentic glomerulonephritis (CrGN), is a severe form of glomerulonephritis and a common presentation of AAV, characterized by the destruction of the renal glomeruli often leading to end-stage renal disease (ESRD) [3]. The diagnosis of AAV relies on serologic studies for ANCA antibodies and tissue biopsy showing pauci-immune vasculitis. This case report highlights the importance of considering hydralazine-induced AAV in patients with unexplained renal decline and positive ANCA testing.

## Case presentation

An 84-year-old female with a past medical history of type 2 diabetes mellitus, myasthenia gravis, and hypertension on hydralazine 100 mg twice a day for over one year presented with generalized weakness. She reported decreased urination but otherwise denied any other symptoms. The physical examination was unremarkable except for elevated blood pressure of 168/73 mmHg. Labs showed an elevated blood urea nitrogen (BUN) of 77 mg/dL and creatinine (Cr) of 5.77 mg/dL (Table 1), demonstrating an acute kidney injury with a baseline Cr of 1.3 prior to admission. Urinalysis revealed hematuria and proteinuria (Table 2). Complete blood count showed a decreased hemoglobin (6.2 g/dL) as shown in Table 3, which improved to 8.5 g/dL after one unit of packed red blood cells (pRBC) was transfused.

**Table 1:** Basic Metabolic Panel (BMP)

	Values	Normal Range
Sodium	138	136-145 mMol/L
Potassium	4.6	3.5-5.1 mmol/L
Chloride	107	98-107 mMol/L
Carbon dioxide	21.4	20.0-31.0 mEq/L
Blood urea nitrogen (BUN)	77	9-23 mg/dL
Creatinine (Cr)	5.77	0.55-1.02 mg/dL
Glucose	157	74-106 mg/dL

**Table 2:** Urinalysis

	Value	Normal Range
Urine color	light-orange	yellow
Urine appearance	turbid	clear
Urine pH	7.0	5.0-7.5
Urine specific gravity	1.010	1.015-1.030
Urine protein	2+	negative
Urine ketones	negative	negative
Urine blood	3+	negative
Urine nitrite	negative	negative
Urine bilirubin	negative	negative
Urine urobilinogen	normal	normal
Urine leukocyte esterase	negative	negative
Urine RBC	>100	negative

**Table 3:** Complete Blood Count (CBC)

	Values	Normal Range
White blood cell count (WBC)	5.5	4.0-10.5 x10^3/uL
Red blood cell count (RBC)	2.17	3.93-5.22 10^6/uL
Hemoglobin (Hgb)	6.2	11.2-15.7 g/dL
Hematocrit (Hct)	20.9	34.1-44.9 %
Mean cell volume (MCV)	96.3	79.4-94.8 fl
Mean cell Hemoglobin (MCH)	28.6	25.6-32.2 pg
Mean cell Hemoglobin concentration (MCHC)	29.7	32.2-35.5 g/dL
Red cell distribution width (RDW)	16.1	11.7-14.4 %
Platelet count (Plt)	208	150-400 10^3/uL
Mean platelet volume (MPV)	10.2	9.4-12.3 fL

Serology was positive for anti-histone antibodies, proteinase 3 (PR3) ANCA antibodies, and myeloperoxidase (MPO) ANCA antibodies (Table 4). Negative anti-nuclear antibodies (ANA), anti-double-stranded DNA (dsDNA) antibodies, and anti-glomerular basement membrane (GBM) antibodies were noted (Table 4). There was decreased complement C3 while complement C4 was normal.

**Table 4:** Immunology/Serology Results ANCA: antineutrophilic cytoplasmic antibody

Serology	Values	Normal Range
Anti-histone antibodies	5.8	0.0 – 0.9 units
Proteinase 3 (PR3) ANCA/c-ANCA antibodies	1.3	0.0 – 0.9 AI
Myeloperoxidase (MPO) ANCA/p-ANCA antibodies	8.0	0.0 – 0.9 AI
Anti-nuclear antibodies	negative	negative
Anti-double-stranded DNA antibodies	2.0	0 – 4.9 iu/mL
Anti-glomerular basement membrane antibodies	<0.2	<1.0 AI
Complement C3	77	90 – 180 mg/dL
Complement C4	15.8	14 – 44 mg/dL

**Figure 1:**
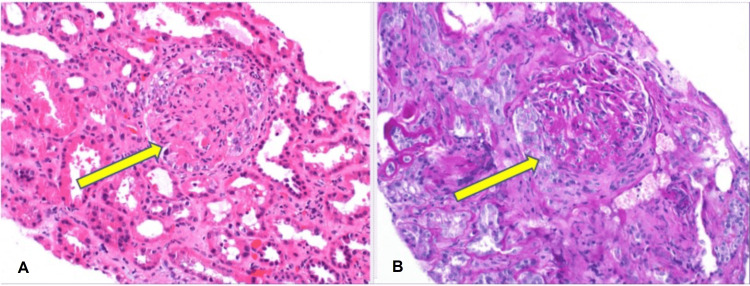
Renal Biopsy (A) Hematoxylin and eosin (H&E) staining with fibrinoid necrosis and cellular crescent, which is indicated by the yellow arrow; (B) Fibro-cellular crescent, which is indicated by the yellow arrow.

A renal biopsy was performed, and pathology showed necrosis and cellular/fibro-cellular crescent formation most consistent with ANCA-associated glomerulonephritis resulting in pauci-immune, necrotizing. and crescentic glomerulonephritis as shown in Figure 1.

Given the above lab results and hydralazine being the suspected triggering factor along with renal biopsy showing pauci-immune, necrotizing, and crescentic glomerulonephritis characteristic of ANCA-associated glomerulonephritis, a diagnosis of hydralazine-induced AAV was made.

Hydralazine was discontinued and the patient was treated with intravenous methylprednisolone 1 gm for three days followed by oral prednisone 60 mg daily. Rituximab therapy was planned, but the patient and her family, after extensive discussion and consideration of potential risks and benefits, opted to decline rituximab therapy due to concerns about potential side effects. The patient was discharged on maintenance prednisone with continuation of hemodialysis and instructed to follow up with nephrology as an outpatient.

## Discussion

Hydralazine-induced AAV is a rare but serious complication of long-term hydralazine therapy [4]. Although the precise mechanism is unknown, it is believed to involve the drug triggering the formation of ANCA, which damages small blood vessels in different organs. Additionally, there is an increase in drug-induced AAV in females, those older than 60, and people who suffer from diabetes, heart failure, or pre-existing renal disease [1,5]. Even though the most likely diagnosis in this case was hydralazine-induced AAV with CrGN, it is crucial to take additional differential diagnoses into account to guarantee an accurate diagnosis and course of therapy. The distinction between anti-glomerular basement disease, idiopathic AAV, hydralazine‐induced lupus nephritis (HILN), and hydralazine‐induced AAV presents a diagnostic challenge for clinicians.

Anti-glomerular basement disease is characterized by the autoimmune development of antibodies towards type IV collagen in the GBM leading to rapidly progressive glomerulonephritis, but there should be evidence of anti-GBM antibodies in serum or histology, which was negative for our patient [6,7]. On the other hand, anti-histone and anti-dsDNA usually are absent in idiopathic AAV [8]. HILN is associated with low levels of C3 and C4 and positive ANA, anti-dsDNA, anti-Smith, and anti-histone antibodies [2,9]. In addition, kidney biopsy in lupus nephritis usually presents with immune complex deposits, endocapillary proliferation, wire loops, and hyaline thrombi [2]. Therefore, a biopsy could help to distinguish between the immunofluorescence patterns of lupus nephritis and hydralazine-induced AAV. Despite the possibility that HILN can present positive ANCA, the diagnosis of AAV was supported by the kidney biopsy's finding of pauci-immune glomerulonephritis rather than immune complex deposits and the absence of ANA, anti-dsDNA, and anti-Smith antibodies.

The initial step in the management of hydralazine-induced AAV should be the discontinuation of hydralazine, which can lead to the resolution of symptoms in mild cases. Treatment of severe cases with renal involvement requires immunosuppressive therapy such as steroids, cyclophosphamide, or rituximab [8]. Even with appropriate medical therapy, patients may still experience differences in their treatment response and progression and/or relapse of kidney disease. However, it is still essential to maintain a high index of suspicion to identify and treat hydralazine-induced AAV as soon as possible.

## Conclusions

Early diagnosis and prompt withdrawal of the offending medication followed by immunosuppressive therapy to prevent further organ damage is crucial for hydralazine-induced AAV. While rituximab and/or cyclophosphamide are often recommended for severe AAV cases, respecting patient autonomy and collaboratively reaching a mutually agreeable treatment plan is vital to improve patient outcomes. This case highlights the importance of holding a high index of suspicion for hydralazine-induced ANCA vasculitis to prevent end-organ damage and initiate the therapy at the earliest.
